# Changes in Sensorimotor Connectivity to dI3 Interneurons in Relation to the Postnatal Maturation of Grasping

**DOI:** 10.3389/fncir.2021.768235

**Published:** 2022-01-27

**Authors:** Alex M. Laliberte, Carl Farah, Kyra R. Steiner, Omar Tariq, Tuan V. Bui

**Affiliations:** ^1^Brain and Mind Research Institute, Department of Biology, University of Ottawa, Ottawa, ON, Canada; ^2^School of Biomedical Engineering, University of British Columbia, Vancouver, BC, Canada

**Keywords:** motor maturation, spinal circuits, sensorimotor integration, development, presynaptic inhibition, dI3 interneurons

## Abstract

Primitive reflexes are evident shortly after birth. Many of these reflexes disappear during postnatal development as part of the maturation of motor control. This study investigates the changes of connectivity related to sensory integration by spinal dI3 interneurons during the time in which the palmar grasp reflex gradually disappears in postnatal mice pups. Our results reveal an increase in GAD65/67-labeled terminals to perisomatic Vglut1-labeled sensory inputs contacting cervical and lumbar dI3 interneurons between postnatal day 3 and day 25. In contrast, there were no changes in the number of perisomatic Vglut1-labeled sensory inputs to lumbar and cervical dI3 interneurons other than a decrease between postnatal day 15 and day 25. Changes in postsynaptic GAD65/67-labeled inputs to dI3 interneurons were inconsistent with a role in the sustained loss of the grasp reflex. These results suggest a possible link between the maturation of hand grasp during postnatal development and increased presynaptic inhibition of sensory inputs to dI3 interneurons.

## Introduction

Spinal circuits for movements are established early in development, as evidenced by movements *in utero*. Spontaneous activity within spinal circuits provides the excitatory drive for these early movements, supplemented by sensory afferents that enter the spinal cord at later stages of embryonic development (Naka, 1964; Ozaki and Snider, 1997; Ladle et al., 2007). Spinal circuits can generate rhythmic and patterned muscle activity underlying locomotion at embryonic stages (Altman and Sudarshan, 1975; Branchereau et al., 2000; Talpalar et al., 2011). However, early movements only display basic features that will become more complex and refined after birth (Dominici et al., 2011; Sylos-Labini et al., 2020). The eventual establishment of descending pathways from the brain and the brainstem to the spinal cord will enable voluntary control of movements (Bareyre et al., 2005; Lemon and Griffiths, 2005; Gu et al., 2017).

As descending inputs are integrated into spinal circuits, the operation of spinal circuits (Perrier and Hounsgaard, 2000; Wilson et al., 2004) and sensorimotor integration continues to be refined in parallel (Betley et al., 2009; Sonner and Ladle, 2013). While sensory inputs are the first to extend into the spinal cord, sensory innervation is continually fine-tuned during postnatal development. During the first three postnatal weeks, sensory connections to the central nervous system are refined, including sprouting and eliminating synaptic inputs (Fitzgerald et al., 1994; Granmo et al., 2008; Lehnert et al., 2021). Simultaneously, the circuits that act to gate sensory feedback are also being established. The gating of sensory feedback to spinal circuits is an important spinal mechanism for controlling smooth and coordinated movement (Fink et al., 2014; Koch et al., 2017). One of these gating mechanisms is presynaptic inhibition through axoaxonic GABAergic innervation of the central terminals of sensory afferents (Eccles et al., 1962; Rudomin, 2009; Lalonde and Bui, 2021). Presynaptic inhibition of primary afferents contacting MNs is evident only after the 1st week of postnatal development (Betley et al., 2009; Sonner and Ladle, 2013). This timing roughly coincides with the arrival and establishment of inputs from sensorimotor cortices to the spinal cord (Curfs et al., 1994; Bareyre et al., 2005; Hsu et al., 2006).

While motor circuits are being refined, rudimentary movements present at birth are replaced by more coordinated and complex movements. Stereotyped reflexive responses to sensory stimuli, also known as primitive reflexes (Fox, 1965; Isakov et al., 1984; Schott and Rossor, 2003; Futagi et al., 2012), progressively dissipate as the control of movements mature. Currently, the mechanisms that attenuate primitive reflexes to make way for more coordinated and skillful movements are not well understood.

Recent work has established that a population of spinal neurons, the dI3 interneurons (dI3 INs), form a sensorimotor circuit integrating cutaneous low-threshold mechanoreceptors and proprioceptors for grasping in the adult (Bui et al., 2013). Curiously, genetic silencing of dI3 INs also led to a marked decrease of the palmar grasp reflex (PGR; Bui et al., 2013), a primitive reflex characterized by flexion of the digits in response to a light touch of the palmar skin (Hooker, 1938; Rushworth and Denny-Brown, 1959). The maturation of the control of the PGR may lead to the refinement of grasping in adults. In light of their developmental involvement in the PGR and grasping, dI3 INs are ideally positioned as candidates for the study of refinement of hand control.

In this study, we investigated whether the disappearance of a primitive reflex could be related to changes in circuitry involving a specific population of spinal neurons. To this end, we quantified the changes in sensory transmission through the development of sensory feedback and inhibitory gating of sensory information. To relate the changes in the connectivity of dI3 INs to the maturation of motor control, we determined the developmental time point at which the PGR disappears. We then show that sensory afferentation onto dI3 INs is constant throughout early postnatal development but that presynaptic inhibition of primary afferents increases during development. Moreover, the developmental time course of the loss of the PGR follows the development of presynaptic inhibition of afferents contacting dI3 INs. These changes in the connectivity of dI3 INs were compared for cervical and lumbar dI3 INs as a means to compare the development of the cervical and lumbar motor function. Our results support the notion that maturation of motor control during early postnatal development is driven by changes in sensorimotor integration by spinal circuits.

## Methods

### Animals

The expression of YFP was driven by the promoter for the homeodomain transcription factor Isl1 was obtained in double transgenic offspring of Isl1^+/Cre^ (The Jackson Laboratory; Stock No. 024242) and Rosa26-lox-stop-lox-YFP (The Jackson Laboratory; Stock No. 006148) mice, henceforth referred to as Isl1:YFP. To temporarily silence dI3 INs, we generated a hybrid line of mice combining the Isl1^+/Cre^, Vglut2^+/Flp^ (The Jackson Laboratory; Stock No. 030212) and CreON-FlpON-hM4Di (The Jackson Laboratory; Stock No. 029040) mouse lines, henceforth referred to as (Isl1/Vglut2)^hM4Di^ mice. Combining these transgenic mouse lines results in the expression of the hM4Di inhibitory DREADD (Designer Receptor Exclusively Activated by Designer Drugs) in dI3 INs and a subset of nociceptors (Bui et al., 2013; Lagerstrom et al., 2010).

All animal procedures were approved by the University of Ottawa Animal Care Committee and conform to the guidelines put forth by the Canadian Council for Animal Care. Animals used in experiments ranged from postnatal (P) 3–25. Both male and female mice were used in this study.

### Genotyping

The following primers were employed in genotyping the transgenic mice. Isl1 Common Forward (GCCACTATTTGCCACCTAGC), Isl1 Wildtype Reverse (CAAATCCAAAAGAGCCCTGTC), Isl1-Cre Reverse (AGGCAAATTTTGGTGTAC), hM4Di Forward (AGTAAGCTTGGG CTGCAGGT), hM4Di Reverse (CATTGACAGGTGTGAAGTTGG), Vglut2 Common Forward (GAAACGGGGGACATCACTC), Vglut2 Wildtype Reverse (GGAATCTCATGGTCTGTTTTG), and Vglut2-Flp Reverse (ACACCGGCCTTATTCCAAG).

### Immunohistochemistry

Mice were transcardially perfused with ice-cold 4% paraformaldehyde, and their spinal cords were removed and postfixed overnight. The spinal cord was cryoprotected in 30% sucrose, transverse sectioned on a cryostat (40–50 μm) and collected as free-floating sections. Sections were washed three times with PBS, blocked for 1 h in PBS with 0.25% triton-X100 (PBST) and 5% normal goat serum, and then incubated overnight at 4°C with primary antibodies in PBST plus 5% serum. The following day, sections were washed three times with PBS and incubated with Alexa Fluor-conjugated secondary antibodies for 3 h at room temperature and washed three final times in PBS. Antibodies used for fluorescent immunohistochemistry were as follows: chicken anti-GFP (1:1,000; Abcam; RRID:AB_300798), guinea-pig anti-Vglut1 (1:2,500; EMD Millipore; RRID:AB_262185), and mouse anti-GAD65/67 (3 μg/ml; Developmental Studies Hybridoma Bank; RRID:AB_2314499). Sections were mounted onto Superfrost slides with Immu-Mount and coverslipped.

### Synaptic Quantification

For quantification of Vglut1+ inputs, perisomatic terminals were quantified on the entire soma and proximal dendrites (10 μm from the soma) on confocal images in the *z*-axis collected by a Nikon A1MP confocal microscope equipped with four single laser lines. Nikon NIS-Elements Viewer (RRID:SCR_014329) was employed for image analysis. Terminals were manually quantified using orthogonal views to confirm apposition onto the cell body. PKC_γ_+/Vglut1+ boutons onto dI3 INs were also counted to estimate the portion of those Vglut1+ inputs that could originate from the corticospinal tract (Mori et al., 1990; Alvarez et al., 2004; Persson et al., 2006). In addition, GAD65/67+ boutons contacting perisomatic Vglut1+ terminals were counted to estimate presynaptic inhibition of sensory inputs to dI3 INs.

Due to the small size of postsynaptic GAD65/67+ boutons and a large number of these boutons in the spinal cord, we used a different method to quantify postsynaptic inhibition of dI3 INs. Images were preprocessed using an ImageJ script. Preprocessing steps consisted of removing the first three and final three *z*-slices in the three-dimensional images and consistent contrast enhancement *via* histogram stretching. After preprocessing, twenty-one images were used to train the Random Forest classifier (Kreshuk and Zhang, 2019) in the Ilastik graphical interface (RRID:SCR_015246). One model was trained to predict if a pixel in the GAD65/GAD67 channel of an image was part of an inhibitory synaptic bouton, while another was trained to predict if a pixel in the YFP channel of an image was part of a neuronal soma. These models made predictions on all pixels in each file. The predictions were represented by a value between 0 and 1 corresponding to each pixel. Predictions were filtered to exclude those with values below 0.7 and 0.5 in the GAD65 and YFP channels, respectively. Groups of spatially adjacent pixels were then collected into objects, which represented distinct GAD65/GAD67+ boutons or YFP+ neurons. To account for background noise in the GFP predictions, we filtered objects to exclude those less than 10,000 voxels in size. The number of overlapping GAD65/GAD67+ boutons on YFP+ neurons objects in each image was then counted and divided by the number of YFP+ neurons. The latter was manually counted in each image. Post-prediction filtering and quantification were done using Cellprofiler (RRID:SCR_007358). The ROUT outlier identification method was performed (*Q* = 0.01) on the resulting bouton count data to limit the effects of potentially spurious outlier results on the analysis.

### Statistical Analysis

Data are reported as mean ± S.E.M., and comparisons were performed using a one-factor ANOVA followed by Tukey’s multiple comparisons test. Repeated measures data from PGR testing were analyzed using a Mixed Effects Model with the Sidak *post-hoc* test. The threshold for significance was set at 0.05. Curve fitting and ROUT outlier identification were performed using GraphPad Prism 9.2.0 (Graphpad Software; RRID:SCR_002798).

### Palmar Grasp Reflex Testing

To test the presence of the palmar grasp reflex, P6-P21 mice received a light touch across the palmar surface of their forepaws with a 1 mm diameter glass capillary tube. Light pressure was applied until the forepaw was slightly displaced. A positive response was noted whenever this touch elicited flexion of the digits and sustained clasping of the digits for a minimum of 2 s. Each mouse received three trials every day from P6 to P21. All animals, Isl1:YFP and (Isl1/Vglut2)^hM4Di^, received a subcutaneous injection of JHU37160 (0.5 mg/kg) which was administered between 20 and 60 min before forelimb grasp reflex testing. This timeframe is supported by electrophysiological studies of JHU37160 activation of DREADD receptors (Bonaventura et al., 2019). Therefore, to limit the separation of the pups from their mother, each pup from a litter was quickly removed and sequentially injected with JHU37160 20 min prior to the onset of palmar grasp reflex testing and immediately placed back in their home cage. The experimenter was blinded to animal genotype prior to the study endpoint. The hindlimb homolog of the palmar grasp reflex, the plantar grasp reflex, was not examined in detail as this reflex was generally less reliably induced in response to mechanical stimulation.

## Results

### Loss of PGR During Development

**Figure 1:**
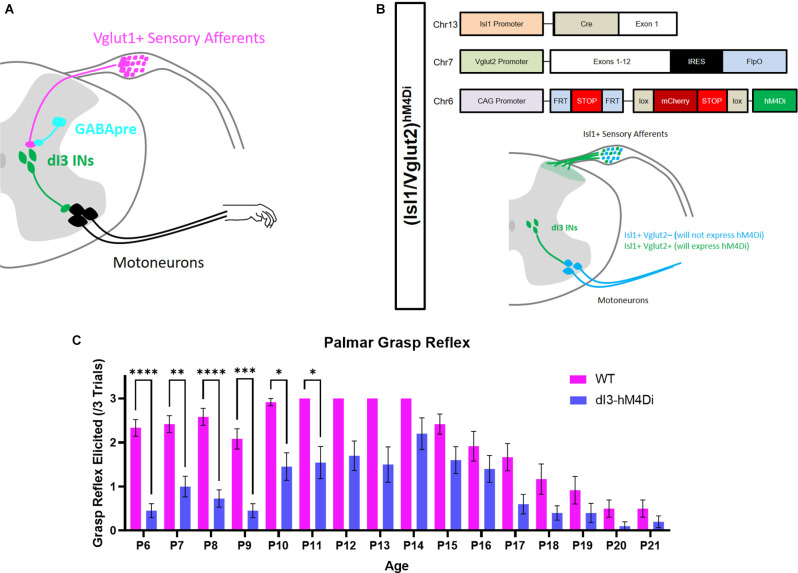
Involvement of dI3 INs in forelimb palmar grasp reflex during postnatal development. **(A)** Putative circuitry involving GABApre presynaptic inhibition of Vglut1+ sensory inputs to dI3 INs. dI3 INs directly excite motoneurons, some of which may be involved in the palmar grasp reflex and mature grasping. **(B)** Diagram of genetics and expression pattern of (Isl1/Vglut2)^hM4Di^ mice. Incidence of the forelimb palmar grasp reflex during postnatal development in **(C)** Isl1:YFP (*N* = 12) and (Isl1/Vglut2)^hM4Di^ (*N* = 10) mice after the administration of the DREADD agonist JHU37160 (subcutaneous 0.5 mg/kg). A statistically significant reduction in the elicitation of the palmar grasp reflex was observed in (Isl1/Vglut2)^hM4Di^ mice (Mixed Effects Model, Sidak’s *post-hoc*). Each animal was tested at every time point. Error bars represent ± S.E.M., ****p < 0.0001, ***p < 0.001, **p < 0.01, *p < 0.05.

We first set out to determine at what developmental time point the PGR disappears. The PGR was elicited in Isl1:YFP transgenic mice from P6 to P21 (Figures 1A,C) using a previously validated method (Bui et al., 2013). Similar to another report of the PGR in mice (Amendola et al., 2004), the incidence of the PGR increased during the 1st postnatal week. The PGR could be consistently elicited in the 2nd postnatal week. After P14, the incidence of PGR declined until P20-P21, after which the PGR was largely absent.

dI3 INs were previously implicated in the grasp reflex using a transgenic approach where these neurons were chronically silenced commencing in embryonic stages (Bui et al., 2013). To circumvent any possible developmental adaptations due to this approach, we expressed hM4Di, a Designer Receptor Exclusively Activated by Designer Drugs (DREADD), in Isl1+/Vglut2+ neurons (Figure 1B). dI3 INs could thus be silenced by JHU37160, a DREADD agonist (Bonaventura et al., 2019). Application of JHU37160 in (Isl1/Vglut2)^hM4Di^ mice reduced the activation of the palmar grasp reflex (Figure 1C; *p* < 0.0001, Mixed Effects Model). These results further support the involvement of dI3 INs in the palmar grasp reflex.

### Development of Primary Afferent Innervation

**Figure 2:**
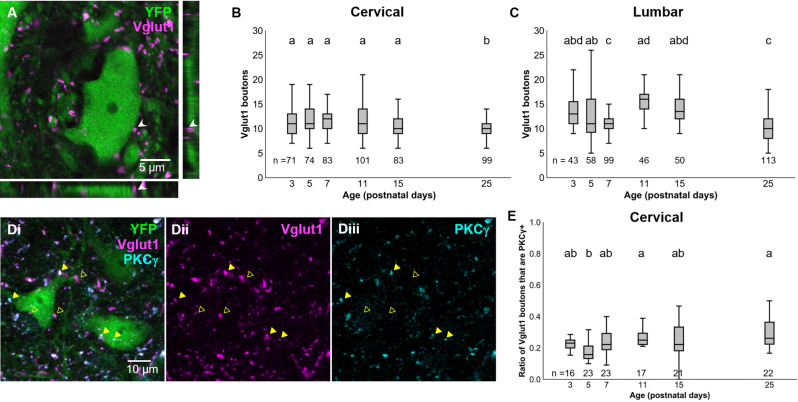
Development of Vglut1+ inputs on dI3 INs. **(A)** Vglut1+ terminals on lumbar YFP+ dI3 IN from a P3 spinal cord. **(B)** Vglut1+ boutons on cervical dI3 INs and **(C)** lumbar dI3 INs at time points between P3 and P25. **(D)** PKC_γ_+/VGLUT1+ (filled triangles) and PKC_γ_-/VGLUT1+ (clear triangles) boutons on cervical dI3 IN from a P25 spinal cord. **(E)** Time course of development of the ratio of PKC+/VGLUT1+ boutons on cervical dI3 INs. One-factor ANOVA. Pairs of groups sharing a letter are not statistically different at the *p* < 0.05 level according to Tukey’s *post-hoc* Test. For example, the means of two groups with the letter “a” are not pairwise statistically different.

Our results above confirm prior observations that the PGR disappears during postnatal development in mice (Amendola et al., 2004; Bui et al., 2013). With the knowledge that dI3 INs are essential for the PGR, we sought to characterize which developmental changes to the sensorimotor circuitry of dI3 INs could contribute to the disappearance of the PGR during maturation. We first asked how sensory inputs to dI3 INs change during development. We identified dI3 INs by yellow fluorescent protein (YFP) expression in Isl1:YFP transgenic mice. Vesicular glutamate transporter 1 (Vglut1) expression has been known to characterize sensory terminals relaying proprioceptive and innocuous mechanoreceptive information (Alvarez et al., 2004) and was employed to identify sensory terminals onto dI3 INs (Figure 2A). The number of perisomatic Vglut1^+^ boutons contacting cervical dI3 INs did not significantly change during development until a decrease at P25 (Figure 2B; *N* = 4 animals per time point, one-factor ANOVA: *p* < 0.0005). The mean number of boutons per cell ranged from 11.2 ± 0.4 (mean ± S.E.M.) to 12.5 ± 0.4 between P3 and P15. It decreased to 10.3 ± 0.2 at P25. The number of Vglut1+ boutons contacting lumbar dI3 INs was lower at P7 and P25 time points (Figure 2C; *N* = 4 animals per time point, one-factor ANOVA: *p* < 0.0001). The mean number of perisomatic Vglut1+ boutons per cell ranged from 13.4 ± 0.8–15.9 ± 0.6 at P3, P5, P11, and P15. At P7 and P25, dI3 INs received on average 11.4 ± 0.3 and 10.3 ± 0.3 boutons per cell, respectively.

A portion of Vglut1+ boutons from the spinal cord originates from the corticospinal tract (CST; Alvarez et al., 2004; Persson et al., 2006). These CST inputs can be demarcated from Vglut1+ sensory afferents by PKC_γ_ staining (Mori et al., 1990). dI3 INs have been shown to receive direct corticospinal inputs (Ueno et al., 2018). We assessed the development of CST inputs to cervical dI3 INs to evaluate any possible influence by the CST on the attenuation of the PGR. The ratio of Vglut1+ inputs to dI3 INs that were PKC_γ_+ was steady at most time points ranging between about one-sixth to under one-third of Vglut1+ boutons (Figures 2D,E; *N* = 4 animals per time point, one-factor ANOVA: *p* < 0.0012). The exception was at P5 (0.18 ± 0.02), where the ratio was smaller than at P11 (0.27 ± 0.02) and P25 (0.30 ± 0.02). These results further support that Vglut1+ sensory boutons to dI3 INs do not undergo any major changes during the first few postnatal weeks.

### Primary Afferents Contacting dI3 INs Receive Presynaptic Inhibition

**Figure 3:**
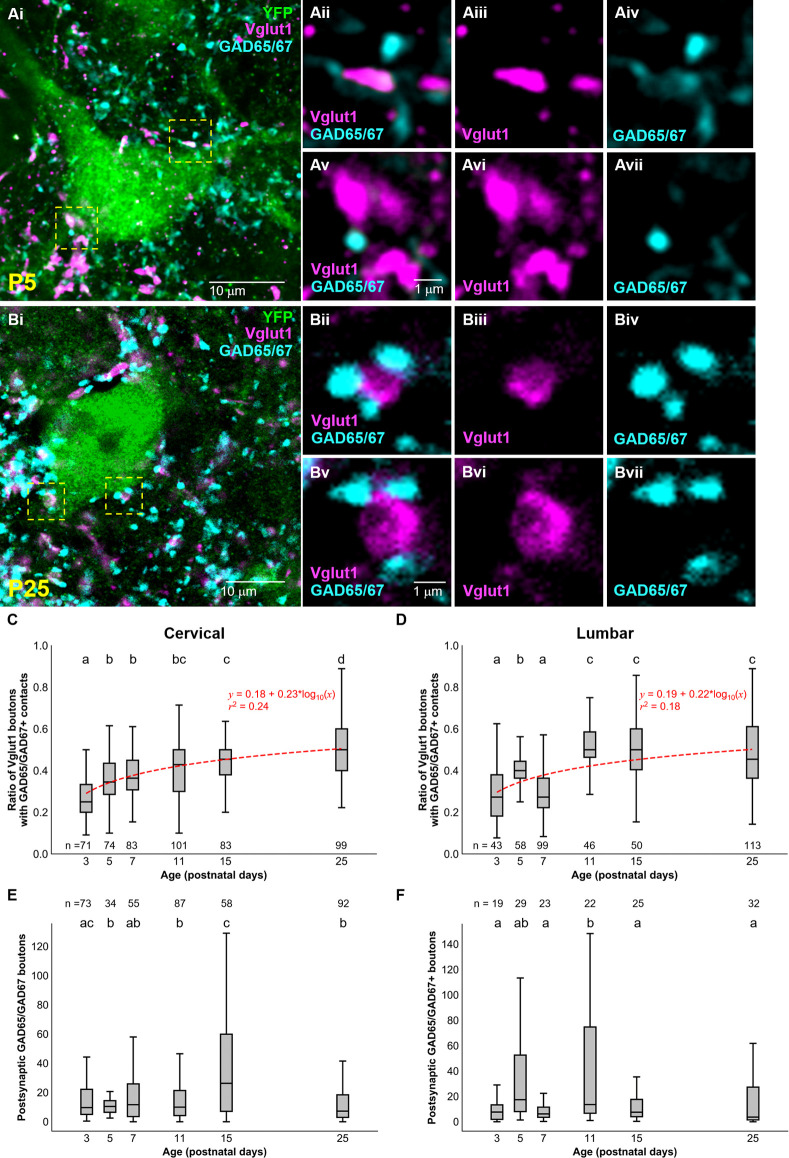
Development of GAD65/67+ boutons on Vglut1+ inputs to dI3 INs. GAD65/67+ boutons contacting Vglut1+ terminals on cervical YFP+ dI3 IN from a **(A)** P5 and **(B)** P25 spinal cord. Boutons in yellow dashed boxes are magnified in Aii-Avii and Bii-Bvii. **(C)** The ratio of Vglut1+ boutons with GAD65/67+ contacts on cervical dI3 INs and **(D)** lumbar dI3 INs at time points between P3 and P25. **(E)** The number of postsynaptic GAD65/67+ boutons per cell on cervical dI3 INs and **(F)** lumbar dI3 INs at time points between P3 and P25. The sample sizes in **(E,F)** refer to the number of images analyzed. A comparison of the means was made using a one-factor ANOVA. Pairs of groups sharing a letter are not statistically different at the *p* < 0.05 level according to Tukey’s *post-hoc* Test. For example, the means of two groups with the letter “a” are not pairwise statistically different. Curve fitting was performed using nonlinear regressions with semilog lines. The 95% confidence intervals for the slopes of **(C,D)** are [0.20, 0.27] and [0.18, 0.27], respectively. The 95% confidence intervals for the *y*-intercept of **(C,D)** are [0.14, 0.22] and [0.14, 0.23], respectively.

Considering that sensory afferentation seemed to remain constant during development, we looked at presynaptic inhibition. Given the importance presynaptic inhibition plays in the execution of smooth movement and grasping (Fink et al., 2014), we asked whether inhibitory axoaxonic connections were present on primary afferents contacting dI3 INs of both the cervical and lumbar spinal cord. We used GAD65/67 as a molecular marker for presynaptic inhibition with the knowledge that GABApre terminals express both GAD65 and GAD67 and contact sensory terminals labeled by Vglut1 while postsynaptic GABAergic terminals solely express GAD67 and are observed on the soma (Betley et al., 2009). To quantify GABApre terminals, we measured the ratio of perisomatic Vglut1+ synapsing onto dI3 INs contacted by GAD65/67+ terminals (Figures 3A,B). From early postnatal development, the ratio of perisomatic Vglut1+ terminals on cervical dI3 INs that were contacted by GAD65/67+ boutons gradually increased from just over a quarter (0.28 ± 0.01) at P3 to over half (0.51 ± 0.01) at P25 (Figure 3C; *N* = 4 animals per time point, one-factor ANOVA: *p* < 0.0001). The development of GABApre terminals to perisomatic Vglut1+ inputs to lumbar dI3 INs followed an almost similar pattern (Figure 3D; *N* = 4 animals per time point, one-factor ANOVA: *p* < 0.0001). The ratio of perisomatic Vglut1+ boutons contacted by GAD65/67+ boutons increased from just over a quarter at P3 (0.28 ± 0.02) to just under half at P25 (0.48 ± 0.02) but with a dip at P7 (0.3 ± 0.01). Curve fitting with a semilog line suggested that the growth in the ratio of Vglut1+ terminals contacted by GAD65/67+ boutons with developmental age was logarithmic in both cervical and lumbar dI3 INs (Cervical: *y* = 0.18 + 0.23*log_10_(x), *r^2^* = 0.24; Lumbar: *y* = 0.19 + 0.22*log_10_(x), *r^2^* = 0.18).

### Development of Postsynaptic GAD65/67 Inputs

Finally, we studied the development of postsynaptic GAD65/67 inputs to dI3 INs. Our results suggest a decrease of perisomatic postsynaptic GAD65/67 inputs to cervical dI3 INs at the initial stages of postnatal development, from P3 (27.5 ± 5.1) to P5 (11.7 ± 1.3), and relatively similar values from P5 onwards with the exception of a notable transient increase from P11 (15.7 ± 1.6) to P15 (37.8 ± 4.8) (Figure 3E; *N* = 4 animals per time point, one-factor ANOVA: *p* < 0.0001). However, postsynaptic GAD65/67 inputs to cervical dI3 INs were significantly reduced from P15 to P25 (12.3 ± 1.3). In the lumbar region, we observed higher postsynaptic GAD65/67 inputs to dI3 INs at P11 (45.9 ± 12.2) compared to P3 (9.0 ± 1.9), P7 (9.2 ± 1.9), P15 (12.1 ± 2.55.0) and P25 (15.3 ± 3.5) (Figure 3F; *N* = 4 animals per time point, one-factor ANOVA: *p* < 0.0001).

## Discussion

Many elements of spinal circuits for movement are already established at birth (Branchereau et al., 2000; Clarac et al., 2004), including specific sensorimotor pathways (Mears and Frank, 1997; Sonner and Ladle, 2013). The maturation of motor control that is present shortly after birth coincides with significant changes in circuitry in the spinal cord: descending pathways continue their establishment of connections with spinal circuits—a process that begins in later embryonic stages (Branchereau et al., 2000), sensory afferent inputs onto spinal circuits are refined through both sprouting and pruning (Fitzgerald et al., 1994; Granmo et al., 2008), the connectivity of some spinal circuits become more specific (Wilson et al., 2004; Bui et al., 2013; Sonner and Ladle, 2013), and the intrinsic properties of spinal neurons continue to mature postnatally (Jiang et al., 1999; Smith and Brownstone, 2020; Sharples and Miles, 2021).

Attempts to ascribe these changes to specific aspects of the maturation of motor control have been relatively lacking. Part of the maturation process of motor control after birth involves the disappearance of primitive reflexes. Primitive reflexes have been known to disappear during the development of the central nervous system and reappear when inhibition is released, a phenomenon observed in patients with cortical lesions (Futagi et al., 2012). Few studies have investigated whether the circuitry employed by primitive reflexes is conserved during development and if these circuits can serve a purpose in a mature nervous system. Previous reflex studies have established that the PGR can be robustly elicited from P7 onwards (Fox, 1965). However, the mechanisms underlying the fading of this particular reflex have not been well documented.

In this study, we focused on the development of grasp control due to the identification of a population of spinal interneurons that is intimately involved with this movement (Bui et al., 2013). We first confirmed using a chemogenetic approach that inhibiting dI3 INs in postnatal mice disrupted the palmar grasp reflex. A caveat of this approach is that the conditional expression of the DREADD receptor hM4Di was based on coincident Isl1 and Vglut2 expression. This intersectional approach restricted DREADD expression not only to dI3 interneurons, but also to a subset of Vglut2+ sensory afferents involved in nociception and pruriception (Lagerstrom et al., 2010; Scherrer et al., 2010). However, the grasp reflex has been ascribed to low-threshold mechanoreception or proprioception (Hooker, 1938; Rushworth and Denny-Brown, 1959), and to the best of our knowledge, nociceptors and pruriceptors have not been involved in low-threshold mechanoreception, even at early developmental stages.

Chemogenetic silencing of dI3 INs effectively attenuated the palmar grasp reflex, albeit the effect of silencing was more pronounced in the earlier time points of the experiment. The relative decline in chemogenetic silencing efficacy aligns with previous findings suggesting that prolonged DREADD inhibition can induce homeostatic synaptic up-scaling (Wen and Turrigiano, 2021) and receptor desensitization (Carvalho Poyraz et al., 2016). Despite this technical limitation, the robust inhibition of the palmar grasp reflex through dI3 IN silencing from P6-P11 provides additional evidence of dI3 IN involvement in the palmar grasp reflex and supports previous observations from permanent loss-of-function studies (Bui et al., 2013).

We then probed for possible changes in sensory connectivity to dI3 INs that may have the same time-course as the disappearance of the grasp reflex. Postnatal refinement of spinal reflexes has been previously reported. For example, the cutaneous flexion withdrawal reflex switches from being driven by low-threshold fibers at early postnatal stages to high-threshold fibers later in development (Fitzgerald et al., 1988). The receptive fields and thresholds of these nociceptive reflexes are modified, while the amplitude and direction of the evoked motor reflex become greater and more specific (Holmberg and Schouenborg, 1996). Some of these changes may be related to modifications in the connectivity of sensory afferents with their target spinal neurons as sensory afferents in the spinal cord and brainstem undergo a substantial amount of postnatal sensory pruning (Gibson and Clowry, 1999; Granmo et al., 2008; Lehnert et al., 2021). This pruning further supports the idea that maturation of motor control is accompanied by a state of higher regulation of sensory excitability.

Thus, we hypothesized that maturation of grasping, which leads to the disappearance of the PGR and the appearance of volitional grasping, may involve a more regimented sensory drive to dI3 INs. Our findings suggest that levels of Vglut1+ sensory afferent inputs to dI3 INs remain constant during the first 2 weeks of postnatal development for both cervical and lumbar segments. This constant afferentation of dI3 INs, in combination with a decreasing latency of excitatory postsynaptic potentials with postnatal age (Bui et al., 2013), goes against the notion of any decline in the sensory drive to dI3 INs postnatally. A caveat of our interpretation of Vglut1+ terminals as proprioceptive and low-threshold mechanosensitive sensory inputs is the presence of Vglut1+ boutons from descending CST pathways (Alvarez et al., 2004; Persson et al., 2006). However, our staining for the CST input marker, PKC_γ_ (Mori et al., 1990), suggests that between 18 and 30% of those Vglut1+ boutons originate from the CST, a ratio that remained constant except for a modest decrease at P5.

Quantitation of postsynaptic GABAergic inhibition identified a transient peak at P15 for cervical dI3 INs and P11 for lumbar dI3 INs. Similar transient peaks have been previously observed in the changes in the density of ventral GAD+ terminals (Sunagawa et al., 2017) and the quantitation of GAD65 and GAD67 mRNA (Ma et al., 1994) in the postnatal rodent spinal cord. The eventual decline of postsynaptic GABAergic inhibition to cervical or lumbar dI3 INs suggests that postsynaptic inhibition is unlikely to factor into the disappearance of the palmar grasp reflex unless it is mediated by increases in glycinergic inhibition or an increase in the strength of inhibitory synapses (González-Forero and Alvarez, 2005).

In contrast, GABAergic terminals onto the perisomatic sensory inputs to dI3 INs increased during the early stages of postnatal development and remained elevated until at least P25. These results are consistent with previous studies that determined that associative molecular markers for presynaptic inhibition such as GAD65/67 are absent at birth and are subsequently upregulated (Betley et al., 2009). This upregulation suggests an increase in presynaptic inhibition of sensory feedback to dI3 INs similar to the gradual gating of neurotransmitter release by presynaptic inhibition observed for the Ia afferent to motoneuron synapse (Sonner and Ladle, 2013).

Presynaptic inhibition is necessary to produce smooth, coordinated movements by the mature nervous system (Fink et al., 2014; Koch et al., 2017). The development of presynaptic inhibition is likely to depend on the integration of descending inputs conveying motor commands. While the establishment of descending inputs starts during embryonic stages (Glover, 1993; Lakke, 1997), further changes are made postnatally (Curfs et al., 1994; Li and Martin, 2001; Bareyre et al., 2005; Hsu et al., 2006). The continued establishment of descending pathways to the spinal cord may influence the development of presynaptic inhibition.

Our data cannot conclusively answer whether the loss of the PGR is solely due to the increased frequency of GABApre contacts on sensory afferents. Critically, not all Vglut1+ inputs to dI3 INs were contacted by GABApre boutons, even at P25 when the PGR was largely absent. However, some of these Vglut1+ terminals devoid of GABApre contacts are likely to arise from CST pathways, which seem to be relatively unaffected by presynaptic inhibition (Jackson et al., 2006). The loss of the PGR is likely to involve changes in descending pathways and continued refinement of the operation of dI3 INs (Bui et al., 2013) and GABApre neurons. Silencing or ablating the GABApre neurons that specifically contact sensory inputs to dI3 INs could further test the contributions of presynaptic inhibition of sensory inputs to dI3 INs to the disappearance of grasp reflexes. Such an approach will require the identification of molecular markers or the localization of this subset of GABApre neurons.

While we did not explicitly study the disappearance of the plantar grasp reflex, the hindlimb homolog of the PGR, because of technical issues related to the reliability of inducing this reflex, this plantar primitive reflex also disappears eventually during postnatal development. We found similar changes in Vglut1+ inputs to lumbar dI3 INs and increases in GABApre boutons to these Vglut1+ inputs. These similarities suggest that the disappearance of the plantar grasp reflex could be associated with circuitry changes in the lumbar segments that resemble those observed with cervical dI3 INs. The investigation of whether increases in GABApre contacts of sensory terminals accompany the disappearance of other primitive reflexes awaits the identification of the spinal circuits that mediate specific reflexes.

## Data Availability Statement

The raw data supporting the conclusions of this article will be made available by the authors, without undue reservation.

## Ethics Statement

The animal study was reviewed and approved by University of Ottawa Animal Care Committee.

## Author Contributions

AL contributed to the data collection, analysis, figure preparation, and writing of the manuscript. CF contributed to the data collection, analysis, and writing of the manuscript. KS contributed to the data collection. OT contributed to data analysis. TB contributed to the analysis, figure preparation, and writing of the manuscript. All authors contributed to the article and approved the submitted version.

## Conflict of Interest

The authors declare that the research was conducted in the absence of any commercial or financial relationships that could be construed as a potential conflict of interest.

## Publisher’s Note

All claims expressed in this article are solely those of the authors and do not necessarily represent those of their affiliated organizations, or those of the publisher, the editors and the reviewers. Any product that may be evaluated in this article, or claim that may be made by its manufacturer, is not guaranteed or endorsed by the publisher.
